# Time to Positivity of Blood Cultures Could Inform Decisions on Antibiotics Administration in Neonatal Early-Onset Sepsis

**DOI:** 10.3390/antibiotics10020123

**Published:** 2021-01-28

**Authors:** Domenico Umberto De Rose, Alessandro Perri, Cinzia Auriti, Francesca Gallini, Luca Maggio, Barbara Fiori, Tiziana D’Inzeo, Teresa Spanu, Giovanni Vento

**Affiliations:** 1 Neonatal Intensive Care Unit, Medical and Surgical Department of Fetus, Newborn and Infant—“Bambino Gesù” Children’s Hospital IRCCS, 00165 Rome, Italy; cinzia.auriti@opbg.net; 2 Neonatology Unit, Department of Woman and Child Health and Public Health, Fondazione Policlinico Universitario “Agostino Gemelli” IRCCS, 00168 Rome, Italy; aleperrix@gmail.com (A.P.); francesca.gallini@fastwebnet.it (F.G.); lucamaggio@me.com (L.M.); giovanni.vento@unicatt.it (G.V.); 3 Dipartimento di Scienze della Vita e Sanità Pubblica, Facoltà di Medicina e Chirurgia, Università Cattolica del Sacro Cuore, 00168 Rome, Italy; 4 Department of Laboratory and Infectious Sciences, Fondazione Policlinico Universitario “Agostino Gemelli” IRCCS, 00168 Rome, Italy; barbara.fiori@policlinicogemelli.it (B.F.); tiziana.dinzeo@policlinicogemelli.it (T.D.); teresa.spanu@policlinicogemelli.it (T.S.); 5 Dipartimento di Scienze Biotecnologiche di Base, Cliniche Intensivologiche e Perioperatorie, Facoltà di Medicina e Chirurgia, Università Cattolica del Sacro Cuore, 00168 Rome, Italy

**Keywords:** newborns, early-onset sepsis, time to positivity, blood culture, c-reactive protein, antibiotics

## Abstract

(1) Background: Empirical antibiotics for suspected neonatal early-onset sepsis are often prolonged administered, even in the absence of clinical signs of infection, while awaiting the blood cultures results. The C-reactive protein is widely used to guide antibiotic therapy, although its increase in the first hours of life is not always evidence of infection. The aim of this study was to evaluate the time to positivity (TTP) of blood cultures (BC) that develop pathogens in our population of neonates and determine whether TTP could safely inform the decisions on empirical antibiotic discontinuation in neonatal early-onset sepsis and reduce the use of unnecessary antibiotics. (2) Methods: We retrospectively collected data of all newborns ≥ 34 weeks admitted to the Neonatal Intermediate-Care Unit at Policlinico “A. Gemelli” University Hospital (Rome, Italy) from 2014 to 2018, with suspected early-onset sepsis (EOS). The TTP was the time in hours from the first BC inoculation to the bacterial growth. We defined as positive BC only those with a pathogenic organism. (3) Results: In total, 103 out of 20,528 infants born in the five-year study period were admitted to our Neonatal Intermediate-Care Unit because of a suspected EOS and enrolled into the study. The mean TTP of pathogenic organisms was 17.7 ± 12.5 h versus 80.5 ± 55.8 h of contaminants (*p =* 0.003). We found ten positive BCs. The TTP of BC was lower than 12, 36, and 48 h in 80%, 90%, and 100% of cases, respectively. CRP levels on admission were similar in infants with a positive and negative BC (*p* = 0.067). The discontinuation of therapy in asymptomatic infants 48 h after initiation would have resulted in a saving of 217 days of antibiotics (31.1% of total days administered). (4) Conclusion: From our data, the TTP of blood cultures that develop pathogens is less than 48 h in 100% of cases. Therefore, in late preterm and full-term infants with suspected EOS, stopping empiric antibiotics 48 h after initiation may be a safe practice to reduce unnecessary antibiotic use, when blood cultures are negative and infants asymptomatic.

## 1. Introduction

Early-onset sepsis (EOS) is a major concern for neonatologists. The most frequent causative pathogens are *group B streptococci* (GBS) and *Escherichia coli* [1]. Currently, neonates with one or more risk factors for EOS, as maternal fever or premature rupture of membranes, undergo sepsis screening, according to the unit protocols, and empirically start antibiotics. Clinicians prolong the therapy even in the absence of clinical signs of infection, while awaiting the results of blood cultures (BC), often on the plasmatic trend of conventional infection biomarkers. New biomarkers such as presepsin (PSEP) seem to be promising to predict EOS [2], but it has not yet entered current clinical practice. Conversely, C-reactive protein (CRP) and procalcitonin (PCT) are the most widely used markers to guide antibiotic therapy, although their early increase after birth is not always related to the onset of infections [3,4]. Recent studies have shown that in cases of suspected EOS, when the neonate has no uncertain symptoms and the blood culture is negative at 36–48 h, the discontinuation of empiric antibiotics, guided by the negativity or reduction of the biomarkers of infection (CRP, PCT, and/or PSEP) is a safe practice and saves improperly used antibiotics and costs, with a low rate of suspected re-infections (less than 1%) but no culture-proven bacterial re-infections [5], and above all, without study-related mortality [5,6,7].

In this, we evaluated the time to positivity (TTP) of BCs that yielded pathogenic microorganisms in our population of late preterm and term neonates and whether it could safely inform the decisions on empirical antibiotic discontinuation in suspected neonatal EOS and reduce the use of unnecessary antibiotics.

## 2. Methods

### 2.1. Design of the Study

We retrospectively collected clinical and laboratory data of all the newborns with a gestational age (GA) higher or equal to 34 weeks, who were admitted from 2014 to 2018 to the Neonatal Intermediate-Care Unit of Policlinico “A. Gemelli” University Hospital (Rome, Italy) because of a suspected EOS. We extracted information through electronical medical records.

According to the Center for disease control and prevention (CDC) 2010 guidelines [8], in our unit, infants with perinatal risk factors (RFs) for infection undergo blood culture and CRP measurement at six to twelve hours of life and start intravenous antibiotics. Empirical therapy continues for at least 72 h; at this time point, blood cultures (BCs) are ne-gative and the baby is asymptomatic.

CRP values were measured using the bedside immunoturbidometric assay Quick-Read CRP (CRP-Q; Orion Diagnostic, Espoo, Finland), requiring about three minutes to obtain the result [9]. Each pediatric bottle (BacT/Alert^®^, bioMérieux, Marcy l’Etoile, France) was inoculated with two ml of the blood sample [10] before the start of antibiotics. Bottles were immediately loaded into a BACT/ALERT^®^3D (bioMérieux) instrument and incubated up to five days or until they signaled positive. When the growth index of a bottle was positive, broth aliquots were collected for standard identification studies, which entailed Gram staining, direct analysis in the Bruker MALDI BioTyper (Bruker Daltonik GmbH, Leipzig, Germany)—the results of which were immediately reported to the patient’s physician, and solid-medium subcultures. After isolation from the cultures, bacteria were identified by MALDI BioTyper analysis [11]. In cases in which the isolate could not be identified (i.e., log (score) value was <2.0), conventional phenotypical tests and/or sequencing of the 16S rRNA gene were performed as previously described [12,13].

In order to ensure a greater accuracy, the results of liquor cultures and urine cultures were also reported.

### 2.2. Definitions

Suspected EOS was defined as the presence of clinical symptoms suggestive of infection (fever or hypothermia, apnea, respiratory distress, reduced movements, jaundice, abdominal distension, feeding problems, hypotension, vomiting or diarrhea, hypoglycemia or hyperglycemia, seizures) and progressive increase of CRP values > 10 mg/L. Confirmed sepsis was defined as positive BC with the presence of clinical symptoms suggestive of infection and the increase of CRP values > 10 mg/L.

TTP was defined as the time from the start of incubation to a positive signal [14]. *Coagulase-negative staphylococci* (CoNS) were considered as pathogenic organisms if the BC was positive and the baby presented clinical symptoms requiring antibiotics for at least five days. Patients BC positive for CoNS, without clinical and laboratory signs suggestive of infection, were defined as contaminated.

### 2.3. Data Analysis

Categorical variables are presented as numbers and percentages while continuous variables are presented as mean ± SD or median [IQR]. Data distribution was evaluated by the Shapiro–Wilk test. Groups were compared with Fisher test, t-test, or Mann–Whitney test as appropriate, using XLSTAT v.2014.5.03: a *p*-value < 0.05 was considered statistically significant.

## 3. Results

**Figure 1:**
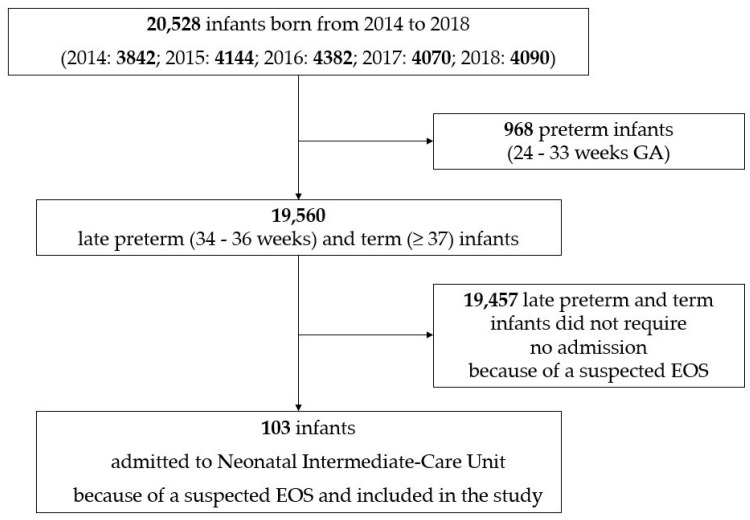
Flowchart of the design of the study.

During the five-year study period, 20,528 infants were born in our III-level hospital. Of them, 103 late preterm and term infants were admitted to our Neonatal Intermediate-Care Unit because of a suspected EOS, started on intravenous antibiotics, and enrolled into the study (Figure 1).

Late preterm and term infants with suspected EOS represented 4.0% of all infants admitted to our Neonatal Intermediate-Care Unit for any reason (103/2603 admissions in the study period).

Table 1 shows clinical characteristics of the study population. We considered BC collected within 72 h of life from each patient. If more than one BC was obtained from the same neonate, we considered only the first. Twelve/103 (11.65%) BC tested positive: four for *group B-Streptococcus* (GBS) (33%), one for *Staphylococcus aureus* (8.3%), and five for CoNS (33%), including two isolates of *Staphylococcus epidermidis*, one of *Staphylococcus haemolyticus*, one of *Staphylococcus warneri*, and one of *Staphylococcus lugdunensis*.

Two positive BCs were defined as contaminated by *Rothia mucillaginosa* and *Micrococcus luteus*.

All twelve isolates recovered from study patients were identified by Bruker MALDI BioTyper at log (score) values > 2.0. Therefore, it was not necessary to use additional tests as 16S rRNA gene sequencing.

Pathogenic organisms had a mean TTP of 17.7 ± 12.5 (max 47) h, while contaminants had a longer mean of 80.5 ± 55.8 (max 120) h (*p* = 0.003).

Considering all twelve identified bacteria, the TTP was lower than 24, 36, and 48 h in 66.7%, 75%, and 91.7% of cases, respectively. Considering only the ten pathogenic organisms, the TTP was lower than 24, 36, and 48 h in 80%, 90%, and 100% of cases, respectively.

No significant differences in CRP levels on admission were found between infants with positive and negative BCs (*p* = 0.067).

We have administered to our patients a total of 698 days of antibiotics: 107 to infants with positive BC and 591 to infants with negative BC, 311/591 (52.6%) days to asymptomatic infants. In these asymptomatic infants, we could have avoided 217/698 (31.1%) unnecessary days of antibiotics and 868 doses of antibiotics, discontinuing the therapy when the BC was negative at 48 h.

Lumbar puncture was performed in 46/103 neonates and liquor culture resulted negative in all cases.

Urine culture was performed in 100/103 neonates, and only six cases resulted positive. Of these, three positive samples (one GBS, one *Escherichia coli*, and one *Enterococcus faecalis*) were found among infants with positive blood culture, and three positive samples (one *Staphylococcus hominis*, one *Escherichia coli*, and one *Enterococcus faecalis*) were found among infants with negative blood culture.

## 4. Discussion

In this study, we found that the mean TTP of BCs yielding pathogenic organisms was 17.7 ± 12.5 (max 47) h, while contaminants needed a longer time. Moreover, in our population of late preterm and term neonates, TTP was lower than 12, 36, and 48 h in the 80%, 90%, and 100% of blood cultures, respectively. Therefore, TTP of BCs could be safely used to guide empiric antibiotic discontinuation, in asymptomatic neonates, when inflammatory biomarkers are negative or in reduction. BCs are the gold standard for the diagnosis of neonatal sepsis. Although the BC positivity rate is often low, being affected by the low blood volume inoculated, prenatal and perinatal antibiotic use, and low-colony count [15], a negative BC at 48–72 h has a high probability of being negative even later. Theodosiou et al. reported in the largest dataset of pediatric blood cultures to date that virtually all clinically significant bacteremias are detectable within 24 h since incubation, without significant differences in TTP for definite pathogens between neonates and older children [16].

The early rise of CRP levels in neonates after the birth usually leads to suspect infections and to start empiric antibiotics, although CRP may increase up to 20 mg/L during the first days of life [17]. We found no significant differences in CRP levels between infants with a positive and negative BC in our study group. According our data, we agree with Macallister et al. that CRP values have a meaning when framed in the context of a patient’s risk factors and clinical symptoms. Therefore, both the start and the length of antibiotic treatment to prevent EOS should not be guided only by CRP measurements [3]. Furthermore, Stocker et al. demonstrated that the negative predictive value of CRP does not increase from 36 h onward [5].

The neonatal early-onset sepsis risk calculator, developed by Kaiser Permanente (California, USA), is increasingly used to predict the risk of EOS in late preterm and term neonates [18] and to decide whether to start antibiotics or not. Its introduction in the clinical practice has reduced antibiotics prescription [19], even if data on its safety are poor [20]. Studies evaluating the use of sepsis calculator to guide empiric antibiotic discontinuation are lacking.

Stopping empiric antibiotics, when the suspicion of EOS is low because of the patient’s good clinical conditions and negative BC, should be the aim to pursue in the management of all infants, to avoid the abuse of unnecessary antibiotics and the onset of antibacterial resistance in microorganisms [21]. Shorter courses of antibiotic therapy are associated with a more rapid recovery from suppression of the gut microbiota [22], whereas prolonged antibiotic exposure has been reported to increase the risk of necrotizing enterocolitis and/or death [23]. In particular, exposure for >10 days resulted in a nearly threefold increase in the risk of developing necrotizing enterocolitis (NEC) [24].

TTP could be a safe strategy to stop antibiotics at a defined time-point in asymptomatic infants. Recently, Kuzniewicz et al. estimated that using a 36 h cut-off compared with 48 h would avoid over 3500 doses of unnecessary antibiotics [7]. We confirmed that 48 h is a safe time-point because in our cohort, a blood culture resulted positive for a GBS only at 47 h, which was probably in relation to maternal antibiotic intrapartum prophylaxis. In the literature, GBS and *E. coli* are usually reported as positive within 36 h in 96–100% of blood cultures, whereas *coagulase-negative Staphylococci* may take up to 48 h to be detected [25].

Our data confirmed that pathogenic organisms had a significantly lower mean TTP, while contaminants had a longer mean TTP, which was in line with Kuzniewicz’s finding [7].

Overall, positive blood cultures by 24 h were found in 66.7% of cases, which is similar to what was previously reported (68%) [7].

The main limitation of our study is the single-center site. It is also well recognized that bottles should be weighed before and after inoculation of blood to ensure an adequate blood volume. A criticism of this work might be that bottles were not weighed before and after inoculation of blood. Although a continuous nursing education was conducted through the study period, we cannot exclude the possibility that the volume of blood drawn could be less than <2 mL in some cases; therefore, the TTP may have been longer. Based on the retrospective design of the study, the a priori number of patients needed to be included into the study was not achieved in the current study. Our study was rather focused on a wide temporal range of five years (2014–2018) to build future quality improvement studies. Furthermore, a reduction in the duration of antibiotic therapy and hospital length of stay should be pursued as an aim and has already been proven as safe in a large sample, with a low rate of re-infections and without study-related mortality [7].

## 5. Conclusions

From our data, the TTP of BCs that develop pathogens is less than 48 h in 100% of cases. Therefore, in late preterm and full-term infants with suspected EOS, stopping empiric antibiotics 48 h after initiation may be a safe practice to reduce unnecessary antibiotic use, when BCs are negative and infants asymptomatic, with inflammatory biomarkers negative or in reduction. Further multicentric studies on a larger population are needed to confirm our data.

## Figures and Tables

**Table 1:** Characteristics of newborns with suspected early-onset sepsis. Data are presented as numbers (%) for categorical variables (compared with Fisher test), mean ± SD or median (IQR) for continuous variables (compared with *t*-test or Mann-Whitney test as appropriate, respectively) as depending on the distribution of the variable.

Characteristics	All Infants (*n* = 103)	Infants with Positive * Blood Culture (*n* = 10)	Infants with Negative ** Blood Culture (*n* = 93)	*p*-Value
Males, n (%)	65 (63)	5 (50)	60 (65)	0.493
Birth weight, median (IQR) (grams)	3280 [2918–3638]	3015 [2806–3580]	3285 [2950–3650]	0.758
Gestational age, mean ± SD (weeks)	39.2 ± 1.6	38.7 ± 1.3	39.3 ± 1.6	0.255
Late preterm infants, n (%)	6 (6)	0	6 (8)	-
Vaginal delivery, n (%)	72 (70)	9 (90)	63 (68)	0.275
Positive maternal vaginal and endoanal swabs, n (%)	29 (28)	3 (30)	26 (28)	0.642
Positive maternal urine culture, n (%)	21 (20)	1 (10)	20 (22)	0.683
Adequate intrapartum antibiotics, n (%)	22 (23)	0	22 (24)	-
Intrapartum fever ≥ 38.0 °C, n (%)	26 (25)	2 (20)	24 (26)	0.227
C-reactive protein value at admission, mean ± SD (mg/L)	27.0 ± 18.2	38.0 ± 24.0	26.6 ± 17.9	0.067
Symptomatic infants, n (%)	48 (47)	5 (50)	43 (46)	0.821
Days of antibiotic treatment, mean ± SD	6.8 ± 2.3	10.7 ± 3.7	6.4 ± 1.5	<0.001
Time to positivity of blood culture (hours) *	-	17.7 ± 12.5 [min: 1.5; max 47]	-	-

* This refers to positive blood culture for pathogenic organisms only. ** This refers to negative blood cultures and to blood cultures positive for the contaminants.

## Data Availability

All considered data in this study are reported in this article.
